# Supplementary material for: Potential Antifungal Effect of Copper Oxide Nanoparticles Combined with Fungicides against Botrytis cinerea and Fusarium oxysporum
Source: Antibiotics (Basel). 2024 Feb 26;13(3):215. doi: 10.3390/antibiotics13030215 (PMC10967597; doi:10.3390/antibiotics13030215)
Supplement: Supplementary file 1 [file antibiotics-13-00215-s001.zip › antibiotics-2846579-supplementary.pdf]

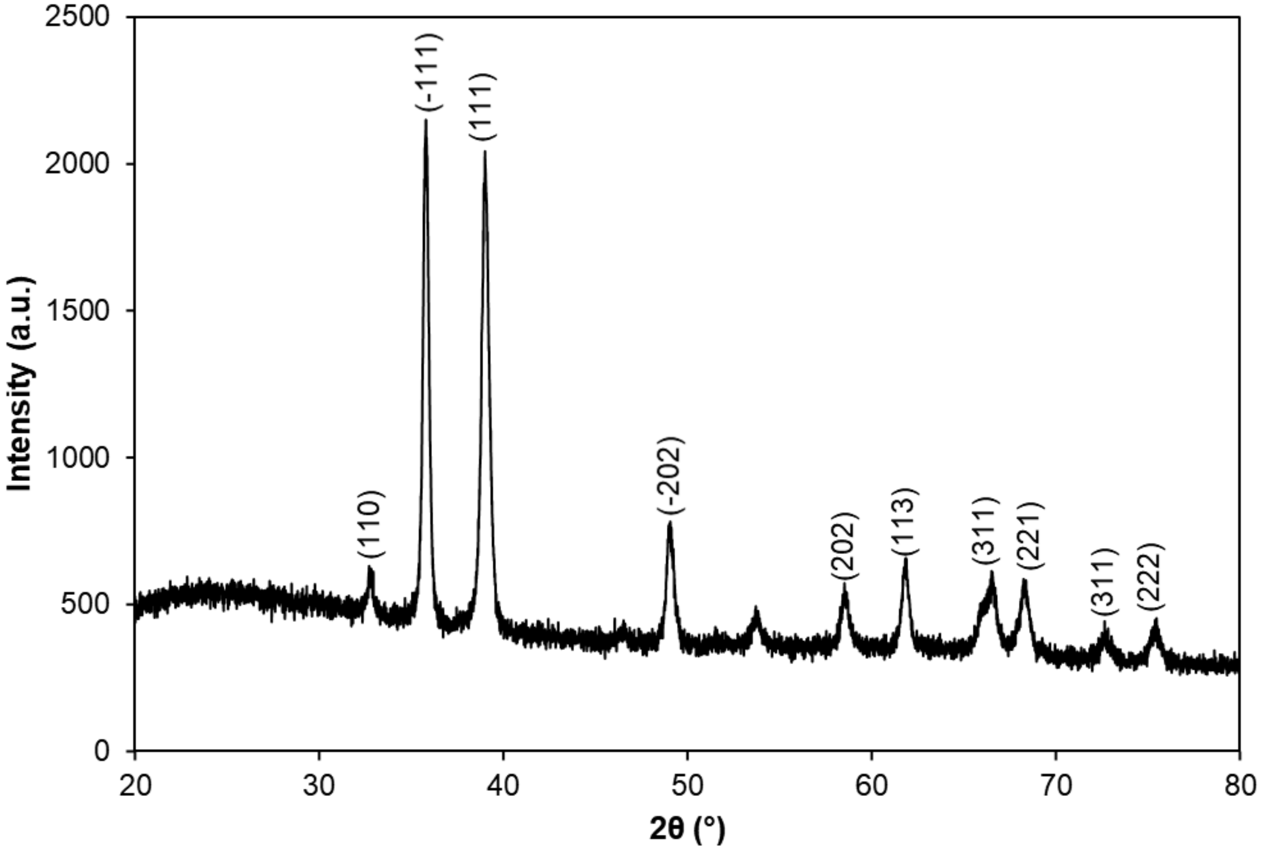

Figure S1. X-Ray diffraction spectrum of Copper Oxide nanoparticles (NCuO).

As shown in Figure S1, Peaks associated to copper oxide were observed at 2θ= 32.9°, 35.9°, 39.1°, 49.1°, 58.6°, 61.9°, 66.5°, 68.4°, 72.8° and 75.5° indexed as (110), (-111), (111), (-202), (202), (113), (311), (221), (311) and (222), corresponding to crystallographic planes of CuO powder matched to reference of the Joint Committee of Powder Diffraction Standard (JCPDS file no. 01-080-0076).
